# Mechanistic insights into the anti-benign prostatic hyperplasia effect of dihydromyricetin via suppression of the 5-AR/TGF-β1/Smad2 axis

**DOI:** 10.1007/s00210-026-04998-3

**Published:** 2026-02-03

**Authors:** Sarah Sameh Abd El-Hameed, Asmaa I. Matouk, Ahmed R. N. Ibrahim, Mahmoud El-Daly

**Affiliations:** 1 Department of Pharmacology & Toxicology, Faculty of Pharmacy, Minia University, Minia, Egypt; 2 Department of Pharmacology and Toxicology, Faculty of Pharmacy, Minia National University (MNU), Minia, Egypt; 3 Department of Biochemistry, Faculty of Pharmacy, Minia University, Minia, Egypt; 4 Department of Pharmacology, College of Pharmacy, King Khalid University, Abha, 62521 Saudi Arabia

**Keywords:** BPH, DHM, PCNA, TLR4, TGF-β1

## Abstract

Benign prostatic hyperplasia (BPH) is a noncancerous prostate enlargement that significantly impacts the quality of life in aged men. Both oxidative stress and inflammation interplay to induce hyperplasia of prostatic epithelial cells as well as seminal vesicle tissues. Dihydromyricetin (DHM), extracted from *Ampelopsis grossedentata*, is used in traditional Chinese medicine for hundreds of years. It exhibited prominent anticancer, anti-inflammatory, and antioxidant activities in several models. To date, its potential protective effects for BPH have not been investigated. The aim of this study was to investigate the role of DHM in the management of testosterone-induced proliferation in prostate and seminal vesicle tissues. Male Wistar rats (200 to 250 g) were divided into three groups: a control group receiving sesame oil (the vehicle), a BPH group receiving testosterone oenanthate (3 mg/kg, subcutaneously) daily for 4 weeks to induce hyperplasia, and a DHM group receiving DHM (50 mg/kg) alongside testosterone oenanthate (3 mg/kg). DHM significantly reduced the prostate and seminal vesicle weights, ameliorated the histopathological changes induced by testosterone, and nearly normalized the serum testosterone, FSH, and LH levels. It also reduced the serum PSA level. Further, DHM reduced MDA levels while increasing GSH levels. Besides, it reduced the prostatic levels of 5-alpha reductase and attenuated TGF-β1/Smad-2 pathway. The anti-hyperproliferation and the anti-inflammatory effects of DHM were attributed to the attenuation of the expression of PCNA, procaspase-3, iNOS, TLR-4, and IL-1β in both the prostate and seminal vesicle tissues. DHM has potential protective effects against testosterone-induced BPH model via downregulation of inflammatory mediators, restoration of oxidative balance, and induction of cell apoptosis.

**Graphical Abstract:**

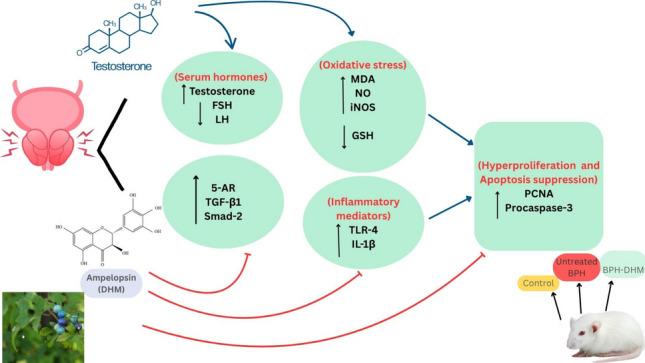

## Introduction

Benign prostatic hyperplasia (BPH) is characterized by a benign growth of the prostate caused by stromal and epithelial cell proliferation. It is prevalent in aging men; nearly 50% of men over 50 years have pathological changes associated with BPH, while those over 80 years have an 80%–90% probability of BPH development (Chughtai et al. 2016). Secondary to prostatic enlargement, individuals with BPH experience symptoms that interfere with their quality of life, such as urgency, nocturia, frequency, urinary incontinence, feeling of incomplete bladder emptying, and post-voiding dribbling (Madersbacher et al. 2019).

Several theories have been proposed to explain how prostate cells proliferate and enlarge, leading to BPH, with the androgenic theory being the most widely accepted. Therefore, 5-alpha reductase inhibitors like finasteride and dutasteride, which inhibit the transformation of testosterone to its active form, have been widely used to manage BPH (Ho And Habib 2011). Unfortunately, patients on such medications often experience adverse effects such as erectile dysfunction, decreased libido, reduced semen, and gynecomastia, which negatively impact compliance and quality of life (Diviccaro et al. 2020). Thus, there is a need to research for alternative therapeutic targets for BPH.

Interestingly, androgens also directly control seminal vesicle proliferation and secretory activity (Okamoto et al. 1982). Testosterone administration in rodents increases seminal vesicle weight and secretions. It also increases the incidence of seminal vesicle carcinoma (Tamano et al. 1996; Dewan et al. 2000; Justulin Jr et al. 2006). Enlarged seminal vesicles compress and block the ejaculatory ducts which in turn may correlate to infertility (Achermann And Esteves 2021).

Oxidative stress plays a crucial role in age-related remodeling of tissue. The progressive peroxidation of cellular lipids by reactive oxygen species (ROS) coupled with the depletion of free radical scavenging enzymes such as superoxide dismutase (SOD), catalase (CAT), and glutathione (GSH) exacerbates oxidative DNA damage, disrupting the balance between cell proliferation and cell death mechanisms and leading to cellular hyperplasia (Choi et al. 2024). Moreover, the proliferating prostate cells consume more oxygen and induce local hypoxia, which further exacerbates massive ROS release, leading to extensive tissue damage (Bostanci et al. 2013) which initiates the secretion of tumor necrosis factor-alpha (TNF-α) and multiple inflammatory interleukins such as IL-8 and IL-1 (Vital et al. 2016; Raafat et al. 2022). In this context, natural products with beneficial antioxidant and anti-inflammatory effects have gained attention in managing BPH (El-Sherbiny et al. 2021; Mitsunari et al. 2021; Raafat et al. 2022).

Dihydromyricetin is a flavonoid obtained from the stems and leaves of *Ampelopsis grossedentata*, *Cedrus deodara*, and *Hovenia dulcis* species. It has been known as a traditional Chinese tea to manage pyretic fever, cough, pharyngitis, laryngitis, and jaundice (Zou et al. 2017). Most recently, it has been used to alleviate alcohol withdrawal symptoms such as anxiety, tolerance, and seizure susceptibility (Shen et al. 2012; Chen et al. 2021). Several studies from our lab demonstrated various pharmacological effects across different animal models through its antioxidant and anti-inflammatory effects. It prevents endothelial deterioration in hyperglycemic settings (Awad et al. 2022), methotrexate induced lung (Matouk et al. 2023), and hepatotoxicity (Matouk et al. 2022), and gentamicin induced nephrotoxicity (Matouk et al. 2024). Moreover, DHM exhibits neuroprotective effects and helps mitigate brain aging in Parkinson’s disease (Ren et al. 2016). DHM exerts its anti-inflammatory activity through the reduction of IL-4, IL-5, and IL-13 cytokines by inhibiting NF-κB signaling pathway and downregulating target genes such as COX-2 and iNOS (Chang et al. 2020; Awad et al. 2022). Furthermore, DHM exhibits anticancer properties, promoting apoptosis and inhibiting cell proliferation via activation of p53 pathway and modulation of NOX4/ROS and TGF-β/Smad signaling pathways (Liu et al. 2015; Xu et al. 2017). Currently, the impact of DHM on BPH has not been well explored.

This study is aimed at investigating the potential protective effects of DHM against testosterone-induced cellular proliferation and pathological changes in prostate and seminal vesicle tissues and at elucidating the underlying mechanisms mediating these effects.

## Materials and methods

### Experimental animals

Adult male Wistar rats (200–250 g) were purchased from the animal care unit of Nahda University in Beni Suef (NUB), Beni Suef, Egypt. Rats were housed for 15 days before the experiment in a 12-h light/dark air-conditioned atmosphere (25 °C). Rats had free access to water and commercial chow throughout the study.

### Drugs and chemicals

Testosterone oenanthate was obtained from Chemical Industries Development Company (CID, Egypt). DHM was purchased from Bulk™ (California, USA). Enzyme-linked immunoassay kits for quantitation of testosterone (Catalog No. 1402020096), LH (Catalog No. 1401030096), and FSH (Catalog No. 1401010096) were obtained from Bios Microwell Diagnostic System (USA). ELISA kits for 5-alpha reductase-1 (5-AR) (Catalog No. ELK7934), transforming growth factor beta-1 (TGF-β1) (Catalog No. ELK2311), Smad-2 (Catalog No. ELK7676), and prostate-specific antigen (PSA) (Catalog No. ELK9631) were obtained from ELK Biotechnology (Colorado, USA). An assay kit for total protein determination was purchased from Biodiagnostic (Cairo, Egypt). Ellman’s reagent, 5,5′-dithiobis (2-nitrobenzoic acid), for reduced GSH determination, 1,1,3,3-tetramethoxypropane (TMP) for preparation of the MDA standard solution, and N-(1-Naphthyl) ethylenediamine dihydrochloride (NNED) for determination of nitrite levels were purchased from Sigma-Aldrich (Saint Louis, USA). For immunohistochemical detection, the primary antibodies against proliferating cell nuclear antigen (anti-PCNA Mouse mAb, Catalog No. GB12010), inducible nitric oxide synthase (anti-iNOS Rabbit pAb, Catalog No. GB11119), and toll-like receptor-4 (anti-TLR4 Rabbit pAb, Catalog No. GB11186) were purchased from Servicebio, Wuhan, China. Antibodies against caspase-3 (anti-caspase-3 Rabbit pAb, Catalog No. YPA 2068) and interleukin-1β (anti-IL-1β Catalog No. YMA 1156) were obtained from Biospes, China.

### Induction of BPH and experimental groups

Rats were randomly divided into three groups (*n* = 8): the control group received sesame oil vehicle (1 ml/kg) for 4 weeks; the BPH group received testosterone enanthate (3 mg/kg/day, s.c.) dissolved in sesame oil for 4 weeks for induction of prostate hyperplasia (Abdel-Naim et al. 2018; Raafat et al. 2022). The BPH-DHM group received testosterone oenanthate (3 mg/kg/day) and DHM (50 mg/kg/day, i.p.) for 4 weeks. The DHM dose was selected based on preliminary work and previous studies (Liu et al. 2017).

### Sample collection

At the end of the experiment, rats were weighed and deeply anesthetized using sodium thiopental (50 mg/kg, i.p.). The loss of the pedal withdrawal reflex confirmed a level of surgical anesthesia. Whole blood was then collected by cardiac puncture. The rats were immediately euthanized following blood collection by decapitation (Al-Odat et al. 2014).

Collected blood samples were centrifuged at 2500 rpm for 10 min to get serum samples. The prostate glands and the seminal vesicles were gently harvested, weighed, and then divided into two halves. The first half was fixed in formaldehyde (10%) for histopathological and immunohistochemical assessments, while the second half was rapidly frozen in liquid nitrogen and stored at − 80 °C for further biochemical analysis.

### Determination of prostate and seminal vesicle indices

Weight index was calculated by dividing the weight of the prostate gland or seminal vesicle (mg) by the animal body weight (g) (Akbari et al. 2021).

### Histopathological examination of the prostate and seminal vesicle tissues

Formalin-fixed tissue samples were dehydrated by ascending concentrations of ethyl alcohol, cleared in xylene, and then embedded into paraffin blocks. Sections of 4–6 μm thickness were stained with hematoxylin–eosin (H&E) (Bancroft And Gamble 2008) and subjected to microscopical examination using a light digital microscope (Olympus XC 30). Histopathological assessment was performed by an expert pathologist blinded to the study groups. The thickness of the epithelial layer was measured at 400 × magnification. The histopathological scoring was performed as previously described (Shackelford et al. 2002). The evaluation is divided into 5 grades as the following: (Grade 1) a score of 1: reflects minimal hyperplasia (< 1%), (Grade 2) a score of 2: reflects slight hyperplasia (1–25%), (Grade 3) a score of 3: reflects moderate hyperplasia (26–50%), (Grade 4) a score of 4: reflects moderate/severe hyperplasia (51–75%), and (Grade 5) a score of 5: reflects severe/high hyperplasia (76–100%).

### Determination of serum testosterone, PSA, FSH, and LH levels

Serum concentrations of testosterone, PSA, FSH, and LH were assessed by ELISA kits according to the manufacturer’s instructions and as previously studied (Pappa et al. 1999; Obisike et al. 2019).

### Assessment of prostatic levels of 5AR, TGF-β1, and Smad-2

The level of 5-alpha reductase 1, TGF-β1, and Smad-2 was also measured in the homogenates of prostatic tissue by ELISA technique following manufacturer’s protocol (An et al. 2020).

### Measurement of oxidative stress parameters

Tissue homogenates (20% w/v) were prepared in cold phosphate buffer saline solution (PBS; 0.05 M, pH 7.4) using a motor-driven homogenizer (LabGEN 7, Cole Parmer, USA). Homogenates were immediately centrifuged for 15 min at 4000 rpm at 4 °C, and the resulting supernatants were separated and kept for the following biochemical analyses.

#### *MDA estimation*

MDA is an indirect indicator of oxidative stress. MDA reacts with thiobarbituric acid (TBA) in the presence of TBA/TCA/HCl reagent, producing a pink color. The color intensity, measured at 534 nm, is proportional to the amount of thiobarbituric acid reactive substances (TBARS) (Senousy et al. 2022).

#### Nitrite estimation

nitrite concentration reflects nitric oxide levels. Nitrite interacts with sulfanilamide (1.0%) and NNED (0.1%), leading to the formation of a purple azo compound measured at 546 nm. The concentration of nitrite was determined using the standard curve (Sun et al. 2003).

#### Reduced GSH estimation

it involves the reduction of Ellman’s reagent (DTNP) by glutathione in tissues, leading to the formation of a yellow compound. The absorbance of this compound, which corresponds to GSH concentration, was measured at 405 nm (Al-Kadi et al. 2020).

### Immunohistochemical assay of inflammatory and apoptotic mediators

Formalin-fixed paraffin blocks of prostate and seminal vesicle tissues were deparaffinized and rehydrated. The sections were then incubated in a 3% hydrogen peroxide (H₂O₂) solution for 20 min to inhibit endogenous peroxidase. After washing Sects. (3 × 10 min) in PBS (0.01 M, pH 7.4), they were incubated in a solution of 0.25% casein in PBS to block nonspecific binding sites for immunoglobulins. Following another PBS wash, sections were incubated with primary antibodies against PCNA (1:500), TLR-4 (1:1000), iNOS (1:500), IL-1β (1:50), and procaspase-3 (1:25) overnight at 4 °C. The slides were then washed (3 × 10 min) in PBS and incubated with a secondary antibody (biotinylated anti-IgG) labeled with a horseradish peroxidase enzyme for 30 min. The respective antigen was visualized by 100 µl 3,3′-diaminobenzidine tetrahydrochloride chromogen solution in 2.5 ml PBS and 50 µl H_2_O_2_ substrate solution, resulting in a brown precipitate. Finally, the tissues were counterstained with hematoxylin.

The immunohistochemical staining was semi-quantitively assessed in five high microscopic power fields (40 ×). The evaluation criteria included the color intensity and the percentage of positively stained cells. The total immunoreactivity score (IRS) of each stained section was examined by a light digital microscope (Olympus xc30, Tokyo, Japan), and the immunostaining intensity values were measured with ImageJ (Version 1.52, USA).

### Statistical analysis

Data were analyzed using GraphPad Prism® (Version 9.00 Windows, GraphPad Software). One-way analysis of variance (ANOVA) followed by the Tukey post-analysis test was used to compare all groups. Data were expressed as mean ± standard error of the mean (SEM), and *p* values less than 0.05 were considered significant.

## Results

### Effect of DHM treatment on prostate and seminal vesicle weight indices

Testosterone treatment for 4 weeks significantly (*p* < 0.05) increased prostate weights as well as seminal vesicle weights of the BPH group compared to the control group, indicating prostate and seminal vesicle enlargement. Treatment with DHM significantly (*p* < 0.05) attenuated the prostate weight and seminal vesicle weight indices (Table 1).

**Table 1:** Effect of DHM treatment on prostate weight, prostate weight index, seminal vesicle weight and seminal vesicle weight index in testosterone-induced BPH

Group	Control	BPH	BPH-DHM
Parameter			
Prostate weight	0.6809 ± 0.0449	1.4260 ± 0.0636^*^	1.1350 ± 0.0776^*#^
Prostate weight index	2.8250 ± 0.2262	6.1930 ± 0.2106 *	4.8060 ± 0.3240 ^*#^
Seminal vesicle weight	0.3054 ± 0.03194	0.8846 ± 0.06615^*^	0.7467 ± 0.02582^*#^
Seminal vesicle weight index	1.652 ± 0.1579	3.802 ± 0.2399^*^	3.133 ± 0.1220^*#^

Data are represented as mean ± S.E.M from 8 rats per group. *Significantly different from control group at *p* < 0.05 and #significantly different from untreated BPH at *p* < 0.05

*DHM* dihydromyricetin, *BPH* benign prostatic hypertrophy

### DHM treatment improves histopathological changes in prostatic and seminal vesicle tissues in testosterone-induced BPH

**Fig. 1:**
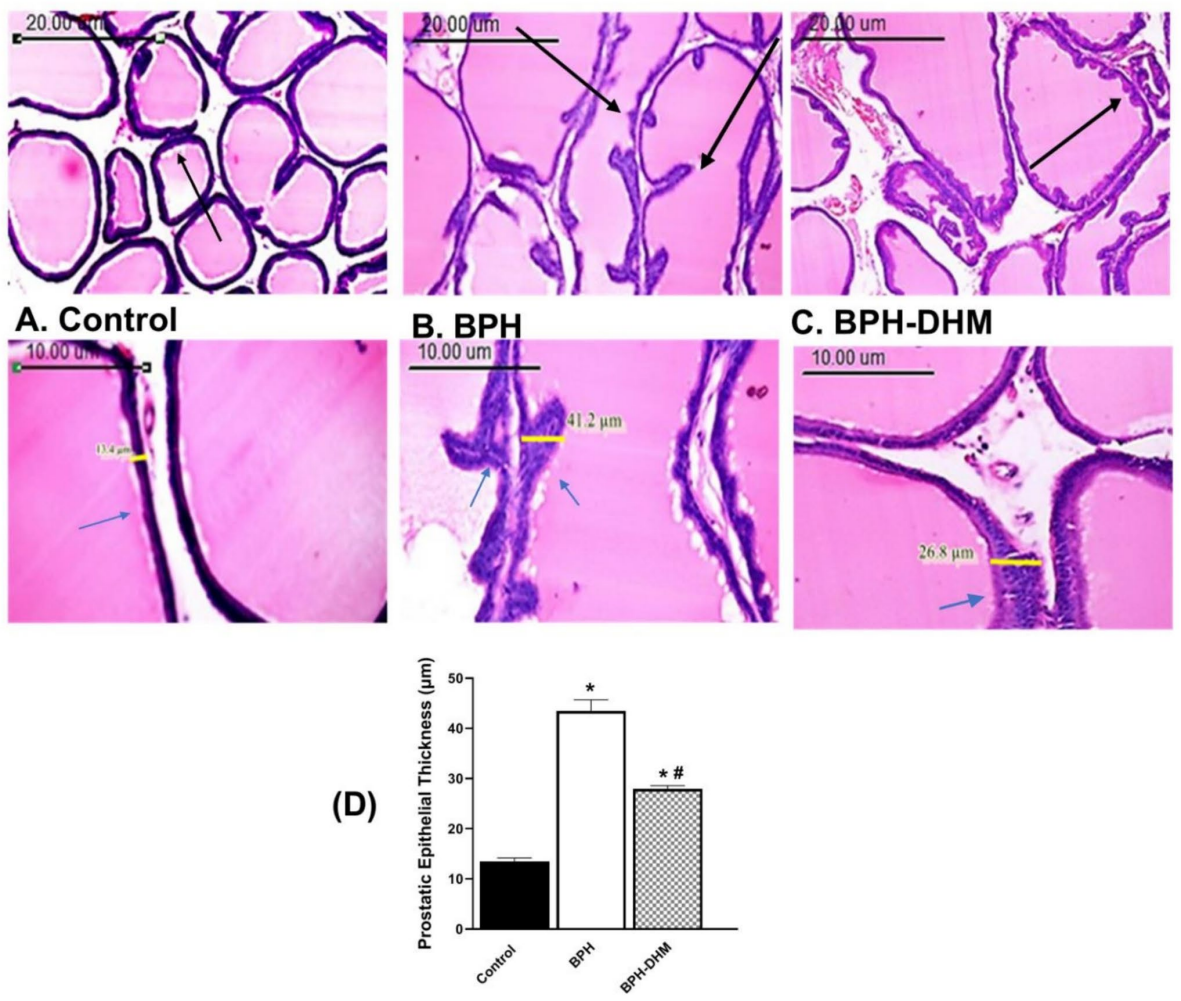
Photomicrographs showing the effect of DHM on histopathological changes induced by testosterone in prostatic tissue. Sections of rat prostate stained with hematoxylin–eosin from the control (**A**), BPH (**B**), and BPH rats treated with DHM (**C**; 50mg/kg, i.p.) for 4 weeks are shown. Upper panel with scale bar = 20 µm - Lower panel with scale bar = 10 µm. **D** Bar chart showing the prostatic epithelial thickness in the three experimental groups. Data are represented as mean ± S.E.M from 8 rats per group. * Significantly different from control group at *p*<0.05 and # significantly different from untreated BPH at *p*<0.05. DHM: dihydromyricetin, BPH: benign prostatic hypertrophy

As expected, the prostate of control rats exhibited normal histological architecture with regular-sized acini and well-organized stroma. The acini were lined by a single layer of cuboidal to tall columnar epithelium that varied in height depending on their secretory activity (Fig. 1A). In contrast, the BPH group displayed irregularly sized and shaped acini with deeply eosinophilic intra-luminal sections. The acini were lined by multiple layers of cuboidal cells. Some of them showed vacuolar degeneration with hyperplastic papillary projections without cellular atypia. Cystic dilatation and thickening of the acinar wall were also noticed (Fig. 1B). Prostatic tissues of rats treated by DHM showed a moderate reduction of acinar epithelial folding in compression with the BPH group. Further, regular size and shape with mild dilatation of the acinar lumen were observed. The thickness of the acinar wall was relatively reduced with focal epithelial hyperplasia in a few acini (Fig. 1C, D and Table 2).

**Table 2:** Quantitative estimation of the histopathology of prostate tissue and seminal vesicle tissues on testosterone-induced BPH model and its alteration by DHM

Group	Control	BPH	BPH-DHM
Parameter			
Prostate	1	4	2
Seminal vesicle	1	3	2

Data are presented as (Grade 1) minimal hyperplasia (< 1%), (Grade 2) slight hyperplasia (1–25%), (Grade 3) moderate hyperplasia (26–50%), (Grade 4) moderate/severe hyperplasia (51–75%), and (Grade 5) severe/high hyperplasia (76–100%). *DHM* dihydromyricetin, *BPH* benign prostatic hypertrophy

**Fig. 2:**
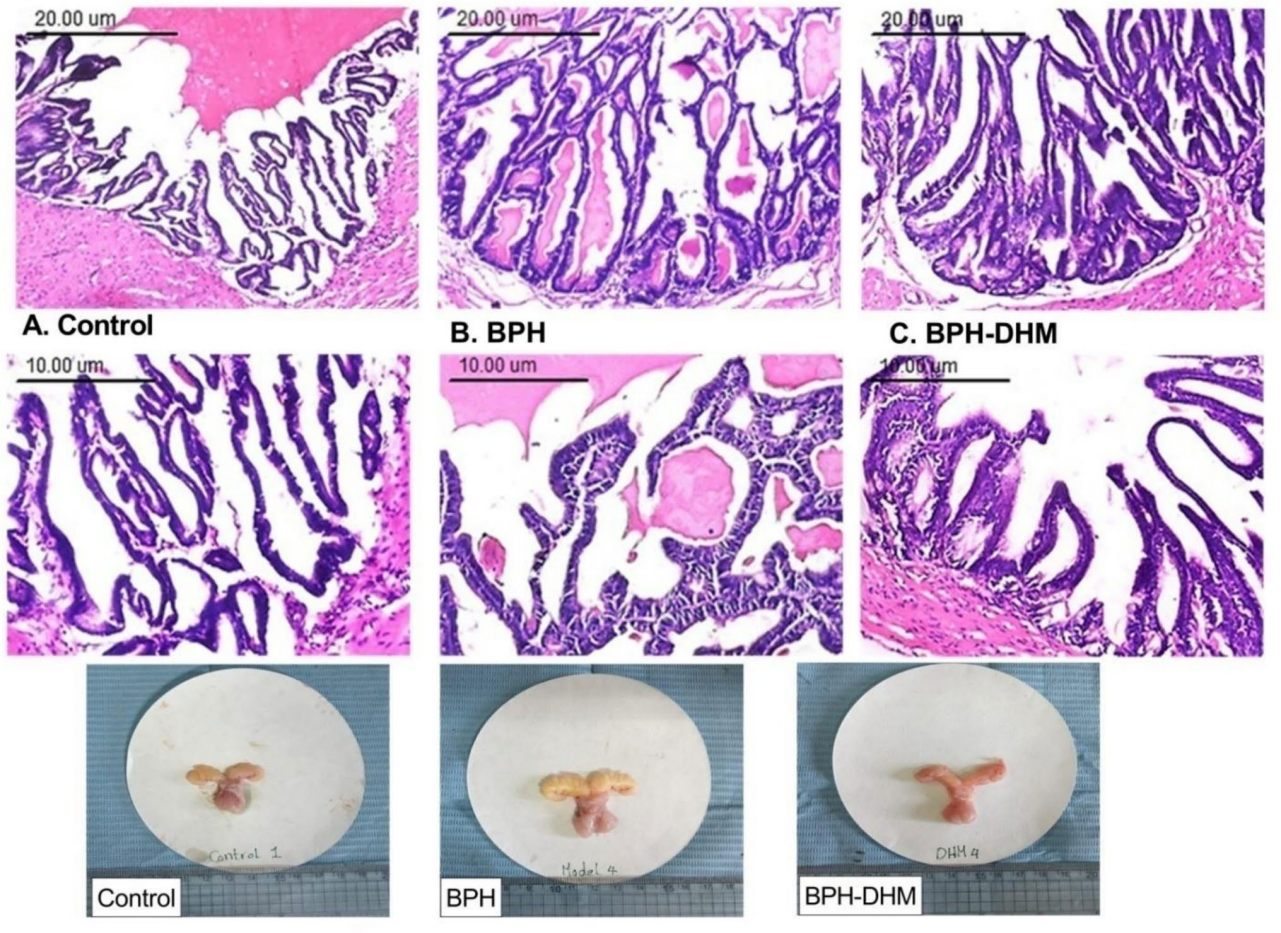
Photomicrographs showing the effect of DHM on histopathological changes induced by testosterone in seminal vesicle tissues. Sections of rat seminal vesicle stained with hematoxylin–eosin from the control (**A**), BPH (**B**), and BPH rats treated with DHM (**C**; 50mg/kg, i.p.) for 4 weeks are shown. Upper panel with scale bar = 20 µm - Lower panel with scale bar = 10 µm. Data are represented as mean ± S.E.M from 8 rats per group. * Significantly different from control group at *p*<0.05 and # significantly different from untreated BPH at *p*<0.05. DHM: dihydromyricetin, BPH: benign prostatic hypertrophy

The examination of the seminal vesicle tissues of control rats revealed sac-like organs with muscular walls surrounding a large central lumen. The mucosa has a honeycombed structure formed by complex folding honeycombed structure to produce irregular anastomosing channels that communicate with the central cavity; thin primary folds of the mucosa also extend out into the vesicle lumen. The epithelium is composed of simple columnar epithelium (Fig. 2A). The seminal vesicle of the BPH group demonstrated disturbance of architecture and diffuse simple hyperplasia of the epithelium with cellular crowding. Degraded secretion in the seminal vesicle was also seen. Folding of the lining epithelium may extend into the alveolar lumina. The epithelial lining of anastomosing channels appeared cuboidal to columnar with increased cytoplasmic basophilia without cellular atypia (Fig. 2B). The seminal vesicle of rats treated by DHM showed multifocal hyperplasia of the epithelial lining without cellular atypia. The glandular structure appeared regular, with an intra-luminal eosinophilic section. The architecture of glandular anastomosing channels revealed improvement compared with the BPH group (Fig. 2C and Table 2).

### Effect of DHM treatment on serum hormone levels and serum PSA

**Fig. 3:**
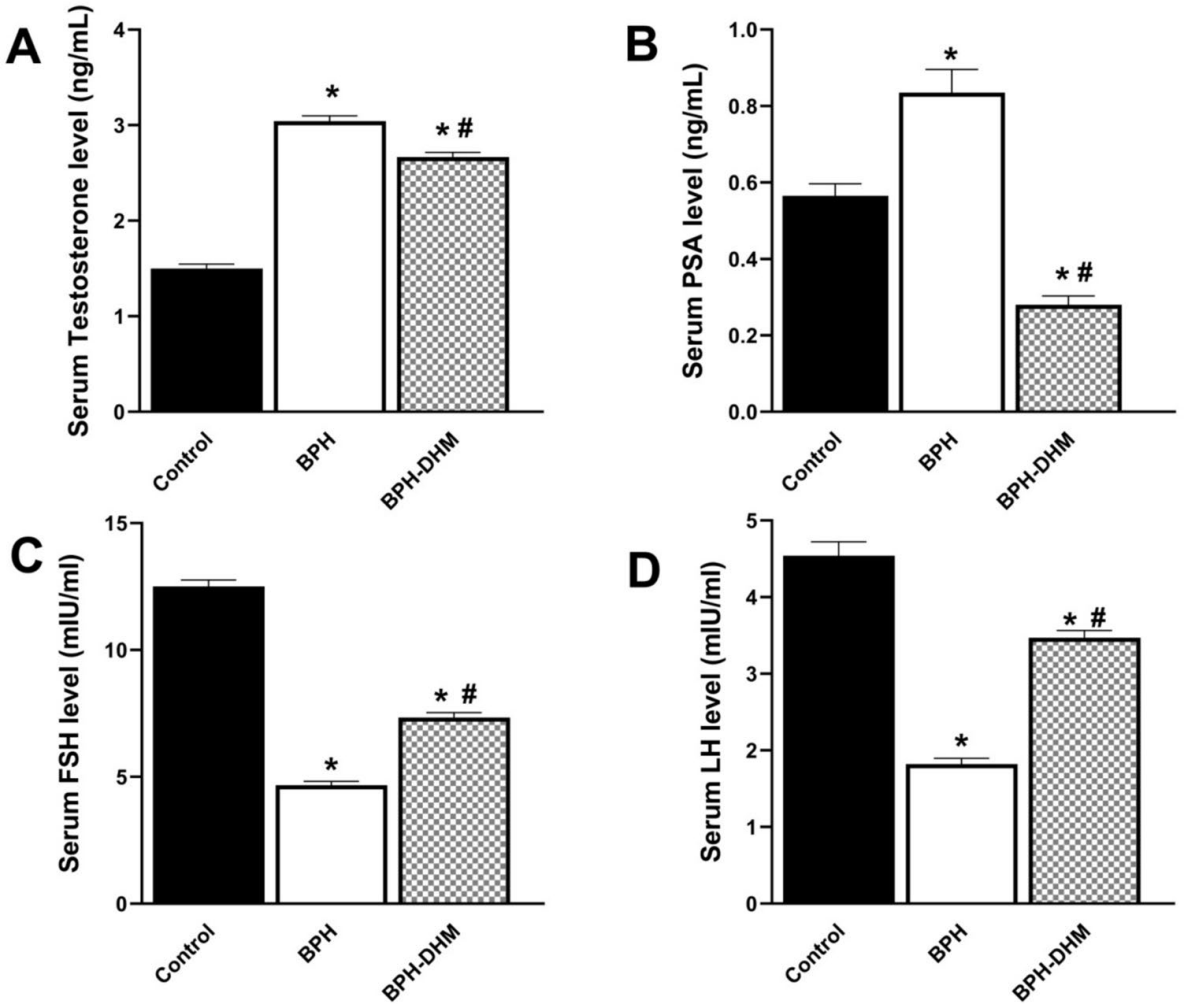
Bar charts showing the effect of DHM treatment on serum hormones levels and serum PSA level in testosterone-induced BPH. **A** serum testosterone, **B** PSA, **C** FSH, and **D** LH. Data are represented as mean ± S.E.M from 8 rats per group. * Significantly different from control group at *p*<0.05 and # significantly different from untreated BPH at *p*<0.05. DHM: dihydromyricetin, BPH: benign prostatic hypertrophy, PSA: prostate specific antigen, FSH: follicle stimulating hormones, LH: luteinizing hormone

Data in Fig. 3 show that serum testosterone levels (Fig. 3A) and serum PSA levels (Fig. 3B) were significantly (*p* < 0.05) elevated in the untreated BPH group compared to the control group. Besides, the serum levels of both FSH (Fig. 3C) and LH (Fig. 3D) were markedly reduced in the BPH rats compared to the normal rats. In contrast, treatment with DHM (50 mg/kg) for 4 weeks ameliorated the elevation of testosterone and PSA serum levels. Moreover, it significantly increased serum FSH and LH levels compared to the untreated BPH group (Fig. 3).

### Effect of DHM treatment on prostatic levels of 5AR-1, TGF-β1, and Smad-2

**Fig. 4:**
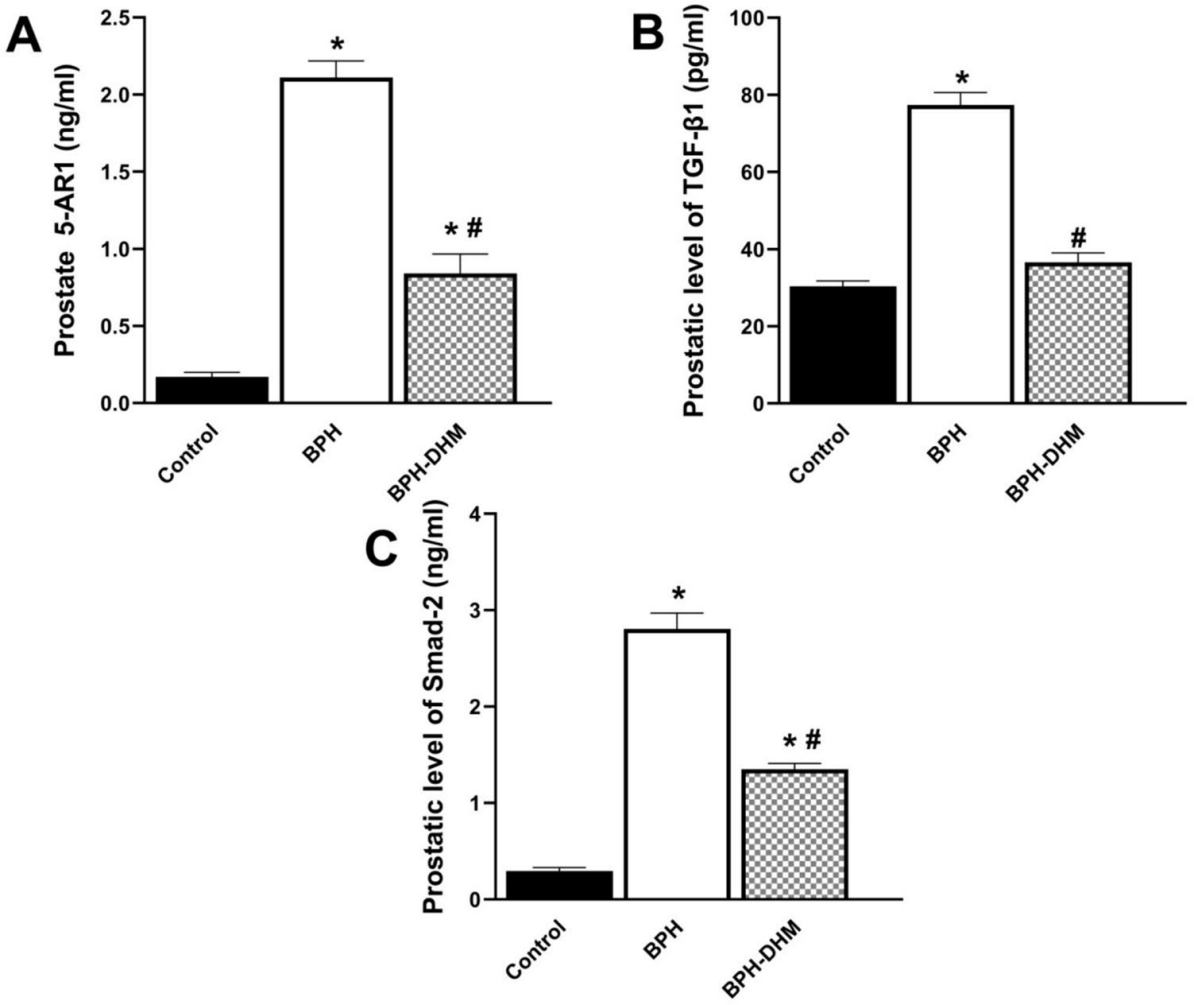
Bar charts showing the effect of DHM treatment on prostatic 5 AR-1 (**A**), TGF-β1 (**B**), and Smad-2 (**C**) levels in testosterone-induced BPH. **C** levels in testosterone-induced BPH. Data are represented as mean ± S.E.M from 8 rats per group. * Significantly different from control group at *p*<0.05 and # significantly different from untreated BPH at *p*<0.05. DHM: dihydromyricetin, BPH: benign prostatic hypertrophy, 5AR-1: 5α reductase-1, TGF- β1: transforming growth factor-beta 1

Figure 4A shows that administration of testosterone significantly (*p* < 0.05) elevated the level of 5AR-1 in the prostatic tissue compared to the control group. Interestingly, DHM significantly (*p* < 0.05) suppressed this elevation compared to the untreated BPH group. Besides, the prostatic levels of TGF-β1 (Fig. 4B) as well as Smad-2 (Fig. 4C) were significantly (*p* < 0.05) elevated in the untreated BPH group compared to the control group. Treatment with DHM significantly ameliorated the rise in their levels.

### DHM treatment alleviates oxidative stress in testosterone-induced BPH model

**Fig. 5:**
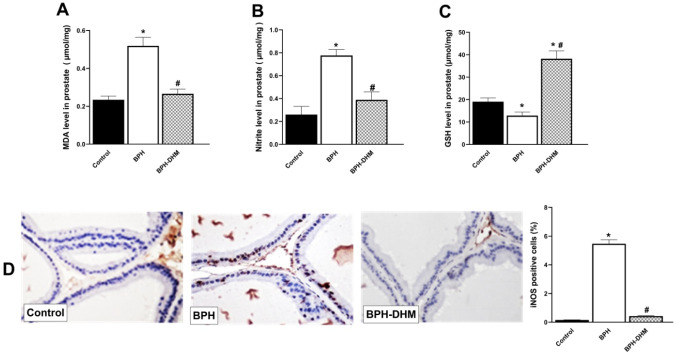
Bar charts showing the effect of DHM treatment on oxidative stress induced by testosterone in prostatic tissues. **A** MDA prostatic level, **B** nitrite prostatic level, and **C** GSH prostatic level. **D** Representative photomicrographs of prostate tissue showing immunohistochemical staining of iNOS and a semiquantitative analysis of its expression on the control group, untreated BPH group, and BPH-DHM group (400 X). Data are represented as mean ± S.E.M from 8 rats per group. * Significantly different from control group at *p*<0.05 and # significantly different from untreated BPH at *p*<0.05. DHM: dihydromyricetin, BPH: benign prostatic hypertrophy, MDA: malondialdehyde, GSH: reduced glutathione, iNOS: inducible nitric oxide synthase

Administration of testosterone triggered oxidative stress in the prostatic tissues, as indicated by the elevated MDA levels (*p* < 0.05, Fig. 5A) and the significant (*p* < 0.05) decline in GSH levels (Fig. 5C). Moreover, as depicted in (Fig. 5B), testosterone significantly (*p* < 0.05) elevated nitrite levels in prostate tissue. This rise in nitrite, the marker of NO radical production, was combined with a significant overexpression of iNOS enzyme in the prostate tissue (Fig. 5D). However, treatment with DHM counteracted the testosterone-induced oxidative imbalance; MDA, NO, and iNOS levels were significantly suppressed, while GSH activity was significantly restored (Fig. 5).

**Fig. 6:**
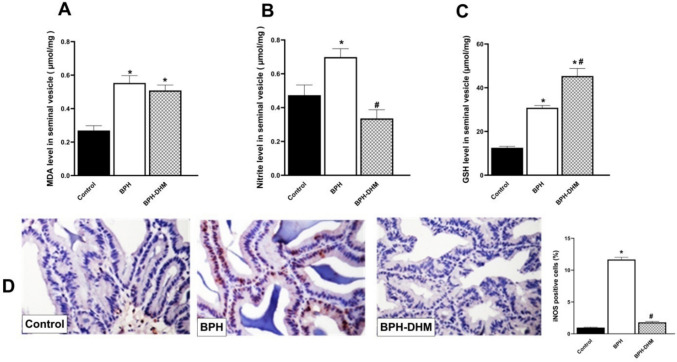
Bar charts showing the effect of DHM treatment on oxidative stress induced by testosterone in seminal vesicle tissues. **A** MDA level, **B** nitrite level, and **C** GSH level, **D** Representative photomicrographs of seminal vesicle tissue showing immunohistochemical staining of iNOS and a semiquantitative analysis of its expression on the control group, untreated BPH group, and BPH-DHM group (400 X). Data are represented as mean ± S.E.M from 8 rats per group. * Significantly different from control group at *p*<0.05 and # significantly different from untreated BPH at *p*<0.05. DHM: dihydromyricetin, BPH: benign prostatic hypertrophy, MDA: malondialdehyde, GSH: reduced glutathione, iNOS: inducible nitric oxide synthase

In comparison with control rats, testosterone administration caused a significant (*p* < 0.05) elevation in MDA levels (Fig. 6A) and a significant (*p* < 0.05) decline in reduced GSH levels in seminal vesicle tissues (Fig. 6C). Total nitrite (Fig. 6B) as well as iNOS expression (Fig. 6D) were also significantly (*p* < 0.05) elevated. Although treatment with DHM significantly abrogated the effects of testosterone on NO and iNOS and reduced GSH seminal vesicle levels, it had a modest effect on MDA level (Fig. 6).

### DHM ameliorates inflammatory and apoptotic markers expression in prostate and seminal vesicle tissues in testosterone-induced BPH

**Fig. 7:**
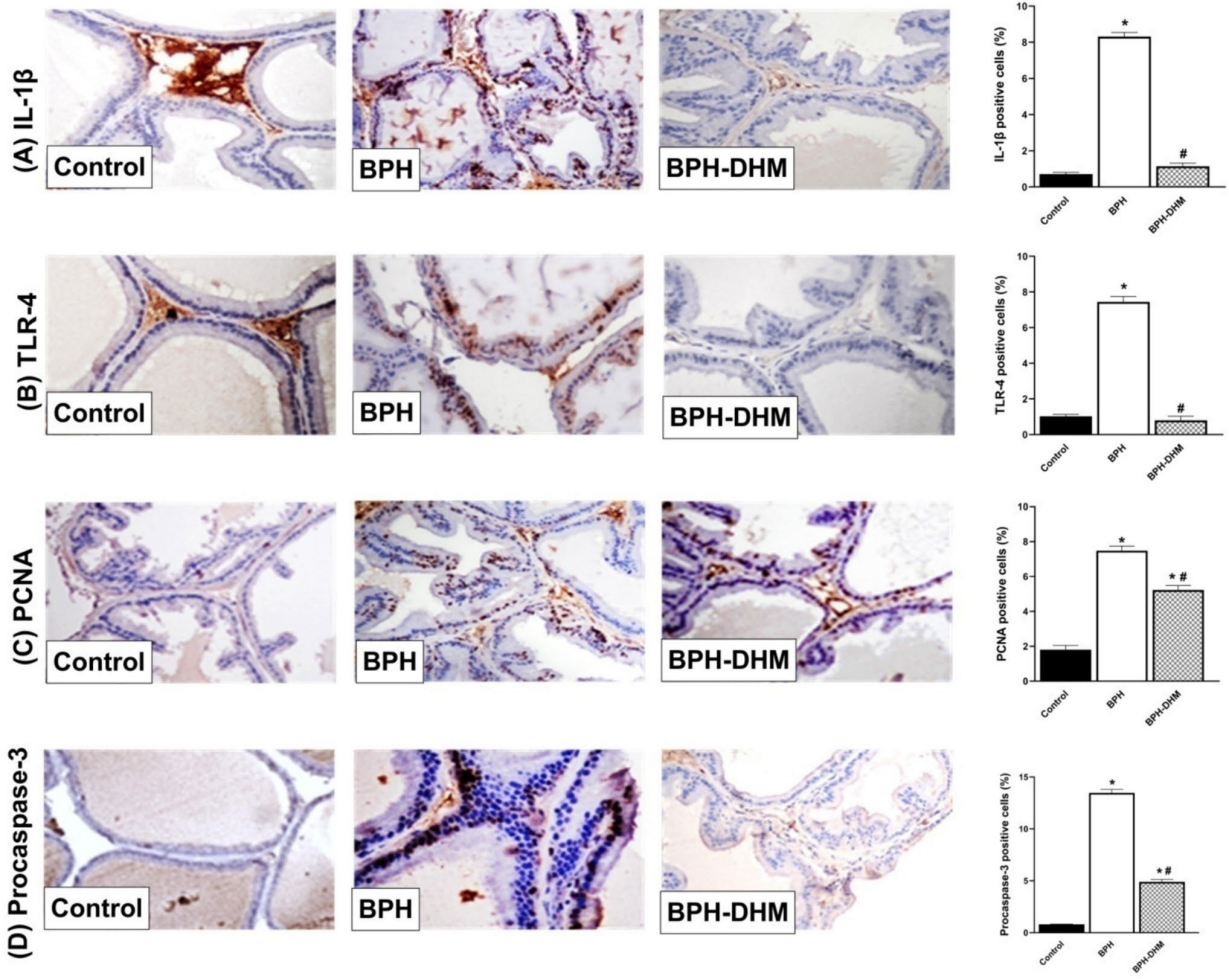
Representative photomicrographs of prostate tissues showing the effect of DHM treatment on the expression of IL-1β, TLR-4, PCNA and procaspase-3 in testosterone-induced BPH. **A** Representative photomicrographs of prostate tissue showing the immunohistochemical staining of IL-1β and a semiquantitative analysis of its expression on the control group, untreated BPH group, and BPH-DHM group (400 X). **B** Representative photomicrographs of prostate tissue showing the immunohistochemical staining of TLR-4 and a semiquantitative analysis of its expression on the control group, untreated BPH group, and BPH-DHM group (400 X). **C** Representative photomicrographs of prostate tissue showing the immunohistochemical staining of PCNA and a semiquantitative analysis of its expression on the control group, untreated BPH group, and BPH-DHM group (400 X). **D** Representative photomicrographs of prostate tissue showing the immunohistochemical staining of procaspase-3 and a semiquantitative analysis of its expression on the control group, untreated BPH group, and BPH-DHM group (400 X). Data are represented as mean ± S.E.M. * significantly different from control group at *p*<0.05 and # significantly different from untreated BPH at *p*<0.05

Testosterone administration provoked a significant (*p* < 0.05) elevation in the prostatic protein expression of key inflammatory markers IL-1β and TLR4, as demonstrated in Fig. 7A and Fig. 7B, respectively. These markers are well-known players with important roles in the initiation and propagation of the inflammatory response within prostate tissue. This contributes to tissue remodeling and hyperplasia observed in BPH. Remarkably, co-administration of DHM with testosterone mitigated this inflammatory response. DHM treatment led to a significant (*p* < 0.05) reduction in the expression of both IL-1β and TLR4 compared to the untreated BPH group, indicating its potent anti-inflammatory action.

In parallel, the prostate tissue from BPH rats showed a robust upregulation in the expression of PCNA, as shown in Fig. 7C. This increase is indicative of enhanced epithelial cell proliferation and supports the previously shown histological data. Additionally, testosterone significantly elevated the expression of the apoptotic marker procaspase-3 (Fig. 7D), suggesting a disrupted balance between survival and cell death within the prostate. Treatment with DHM significantly (*p* < 0.05) reduced the expression of both PCNA and procaspase-3 compared to the untreated group, suggesting a dual modulatory effect of DHM in balancing uncontrolled cell proliferation while restoring apoptotic signaling pathways.

Despite the improvement seen with DHM, the expression levels of IL-1β, TLR4, PCNA, and procaspase-3 in the DHM-treated group remained significantly (*p* < 0.05) higher compared to the control group.

**Fig. 8:**
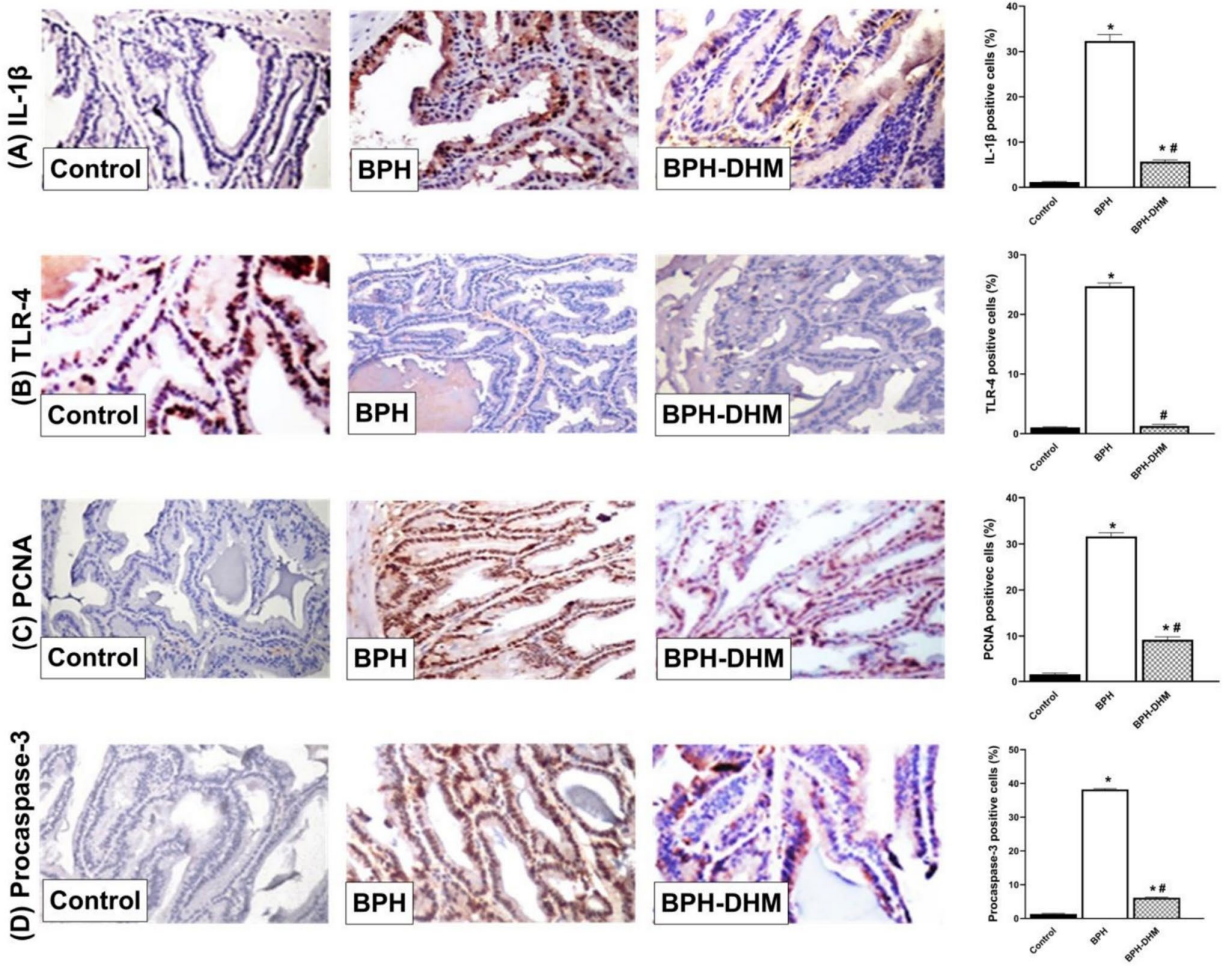
Representative photomicrographs of seminal vesicle tissues showing the effect of DHM treatment on the expression of IL-1β, TLR-4, PCNA and procaspase-3 in testosterone-induced BPH. **A** Representative photomicrographs of seminal vesicle tissue showing the immunohistochemical staining of IL-1β and a semiquantitative analysis of its expression on the control group, untreated BPH group, and BPH-DHM group (400 X). **B** Representative photomicrographs of seminal vesicle tissue showing the immunohistochemical staining of TLR-4 and a semiquantitative analysis of its expression on the control group, untreated BPH group, and BPH-DHM group (400 X). **C** Representative photomicrographs of seminal vesicle tissue showing the immunohistochemical staining of PCNA and a semiquantitative analysis of its expression on the control group, untreated BPH group, and BPH-DHM group (400 X). **D** Representative photomicrographs of seminal vesicle tissue showing the immunohistochemical staining of procaspase-3 and a semiquantitative analysis of its expression on the control group, untreated BPH group, and BPH DHM group (400 X). Data are represented as mean ± S.E.M. * significantly different from control group at *p*<0.05 and # significantly different from untreated BPH at *p*<0.05. DHM: dihydromyricetin, BPH: benign prostatic hypertrophy, IL-1β: interleukin 1β, TLR-4: toll-like receptor-4, PCNA: proliferating cell nuclear antigen

On the other hand, the expressions of IL-1β (Fig. 8A) and TLR4 (Fig. 8B) were significantly (*p* < 0.05) elevated in the seminal vesicle tissues of the untreated testosterone group compared to the control group, and DHM administration significantly mitigated their expressions. Moreover, testosterone induced a significant elevation in the expression of PCNA (Fig. 8C) and procaspase-3 (Fig. 8D) in seminal vesicle tissues. However, these effects were significantly (*p* < 0.05) antagonized by DHM.

## Discussion

The present study provides the first evidence for the anti-proliferative effects of DHM in prostate glands and seminal vesicle tissues via suppressing oxidative stress, inflammation, and cellular proliferating signaling pathways. The concurrent administration of DHM (50 mg/kg) with testosterone reduced prostate as well as seminal vesicle size, restored endocrine balance, reduced serum PSA, and ameliorated histopathological changes induced by testosterone. Some previous studies employed castrated rats in a testosterone-induced BPH model to eliminate endogenous testosterone production and minimize hormonal variability (Kim et al. 2015; Akanni et al. 2020). In this study, BPH was deliberately induced in non-castrated rats to better simulate the clinical condition in humans, where androgenic activity persists during disease development and progression. This approach enhances the clinical relevance of the model by maintaining physiological androgen–prostate interaction. Moreover, the inclusion of the control group ensured that the observed changes were attributable to the exogenous testosterone administration rather than baseline hormonal effects. This methodology adheres to established and validated models for evaluating therapeutic interventions in BPH (Ammar et al. 2015; Jeon et al. 2017; Sasidharan et al. 2022).

Testosterone and its metabolite dihydrotestosterone (DHT) control the growth and function of the accessory male glands, prostate and seminal vesicle (Okamoto et al. 1982; Justulin Jr et al.^2006^; Abdel-Naim et al. 2018; Raafat et al. 2022). In this study, continued testosterone administration for 4 weeks elevated serum testosterone, while FSH and LH levels declined. In response to elevated testosterone levels, the hypothalamus reduces GnRH release, subsequently reducing FSH and LH release from the pituitary gland (Onyegeme-Okerenta et al. 2022). Affecting hypothalamic-pituitary–gonadal axis may lead to a partial or complete cessation of spermatogenesis by reducing LH and FSH (Fusco et al. 2021). Indeed, DHT binds with high affinity to androgen receptors and stimulates growth factors such as epidermal growth factor (EGF) and transforming growth factor-beta (TGF-β). This, in turn, promotes epithelial and stromal cell proliferation, leading to hyperplasia (Gur and Timurkaan 2016; Khan et al. 2025). 5-Alpha reductase-1, which is responsible for the conversion of testosterone to 5-DHT, was elevated in the prostatic tissue of untreated rats. In accordance with this finding, we reported an increase in prostate and seminal vesicle weight indices in the BPH group, indicating hypertrophy in these tissues. These observations were further confirmed by the hyperplastic projections, loss of acinar architecture, vacuolar degeneration, and thickening of the acinar wall as evident in the histopathological examination.

An elevation of serum PSA was observed in parallel with hormone imbalance, prostatic hypertrophy, and histological damage in the untreated BPH group. PSA is a glycoprotein secreted by prostatic epithelial cells and is commonly used as a biomarker for the assessment of BPH. Its expression is primarily attributed to enhanced DHT formation, AR activation, and subsequent hyperproliferation of prostatic epithelium (Zhu et al. 2003; Pejčić et al. 2015; Vickman et al. 2020). Accordingly, the elevated serum PSA recorded in our study confirms the induction of prostatic hyperplasia (Kim et al. 2017; Sasidharan et al. 2022).

The natural polyphenol DHM has a variety of pharmacological actions, mainly correlated to its antioxidant and anti-inflammatory properties (Zhang et al. 2021a, b; Awad et al. 2022; Matouk et al. 2023). In this study, concurrent treatment with DHM counteracted the testosterone-induced gross and histopathological changes in the prostate glands and seminal vesicle tissues. Specifically, DHM reduced the thickness of the prostate epithelial layer and restored the normal acinar lumen structure. Further, DHM improved the glandular structure of the seminal vesicles and reduced their weights. DHM treatment resulted in a small but significant reduction in testosterone level which might explain its effect on pituitary hormones, suggesting a modulatory role of DHM on the hypothalamic-pituitary–gonadal axis that maintained hormonal balance and antagonized testosterone-induced hyperplasia. Restoring hormonal balance and reducing seminal vesicle size may help attenuate impaired spermatogenesis and infertility induced by testosterone. Including semen analysis and assessment of sperm motility and sperm count would strengthen our results. Unfortunately, this data was not available. We will take this into consideration in future studies.

Interestingly, DHM reduced the elevation of 5α reductase enzyme which is expected to suppress the conversion of testosterone to its active metabolite and hence preventing its proliferative effect, which was also confirmed by the reduction of serum PSA. We hypothesized that the inhibitory effect of DHM on 5-alpha reductase enzyme is linked to its chemical structure. DHM is a polyphenol and has structure activity relationship to other compounds including myricetin, quercetin, and fisetin. These compounds were found to inhibit prostate cell hyperplasia by inhibiting 5-alpha reductase activity (Ye et al. 2018; Pejčić et al. 2019; Azizi et al. 2021). However, future studies are required to support this hypothesis. It is important to note that the DHM-treated group still showed higher levels of testosterone when compared with the normal control. This indicates that the ability of DHM to reduce 5-alpha reductase might be linked to the observed improvement in the prostate pathology as this enzyme is important to convert testosterone to its active form which is responsible for prostatic hyperplasia. Measuring DHT would confirm our explanation; however, this was not available and is one limitation of this study.

Additionally, the TGF-β1 signaling pathway plays a crucial role in cellular hyperproliferation and BPH pathogenesis. Several studies reported its overexpression in BPH as well as prostate cancer. Upregulation of TGF-β1 induces phosphorylation of Smad-2, a key downstream mediator that translocates to the nucleus and activates the epithelial-mesenchymal transition (EMT), a key process to the pathogenesis of prostatic hyperplasia in which the immotile epithelial cells transform into motile mesenchymal cells and cause hyperproliferation of the stromal layer (Abdel-Naim et al. 2018; Khan et al. 2025). Interestingly, DHM reduced the prostatic level of TGF-β1 by suppressing 5AR-1 activity and inhibiting DHT formation; these findings were confirmed by the downregulation of Smad-2. It was mentioned that DHM arrested the EMT process and promoted apoptosis via suppressing TGF-β1/Smad-2 signaling pathway (Liu et al. 2015; Huang et al. 2017; Ye et al. 2019).

Previous research associated BPH development with increased oxidative stress (Kayode et al. 2020; Vafa et al. 2020), which comes in line with the marked increases in prostatic and seminal vesicle MDA levels and decreased GSH in untreated BPH animals in this study. MDA is formed as a result of lipid oxidation and serves as an indirect indicator of excess ROS production. It is a toxic substance that diffuses through cells, causing damage to cellular membranes, proteins, and DNA (Minciullo et al. 2015; Vafa et al. 2020). In contrast, GSH is responsible for detoxifying free radicals and peroxides (Njälsson And Norgren 2005). It was reported that high testosterone levels induce cellular metabolism in the hypertrophic tissues leading to increased oxidative stress (NC Tam et al. 2003). Further, the proliferating cells increase oxygen demands and induce local hypoxia that further drives ROS production and tissue damage (Nickel et al. 2008; Bostanci et al. 2013). Additionally, in the BPH group, the expression of iNOS was increased and consequently caused excessive production of NO, a nitrosative stress-related parameter. NO mediates DNA damage directly or indirectly via the generation of a highly oxidizing radical, peroxynitrite (Gradini et al. 1999). Moreover, it induces the antiapoptotic, Bcl-2, gene expression which contributes to cellular hyperproliferation (Minciullo et al. 2015). Treatment with DHM ameliorated testosterone-induced oxidative stress in the current study. It markedly attenuated MDA levels, enhanced GSH activity, and downregulated iNOS expression and NO levels. These findings were compatible with the decreased prostate and seminal vesicle weights and normalized histological organization of these tissues. Interestingly, DHM preserved the male reproductive activity and restored the serum testosterone levels in heat-stressed experimental animals (Yang et al. 2024). Recent research suggested the inhibition of NADPH oxidase (NOX)-dependent ROS generation and upregulation of Nrf2 as possible antioxidant mechanisms of DHM (Wei et al. 2022a, b; Matouk et al. 2023). Nrf2 augments the expression of antioxidant enzymes, including SOD and catalase, and enhances cellular defense against xenobiotic insults (Zhang et al. 2021a, b; Choi et al. 2024).

Another pivotal participant in the development of BPH is chronic inflammation. The inflammatory injury within the prostate tissues stimulates the production of multiple cytokines and local growth factors that induce tissue remodeling and stromal hyperproliferation (Chughtai et al. 2011; Ribal 2013; Fujii et al. 2019). TLR-4-mediated signaling is a key inflammation switch in the immune system in response to infection, tissue injury, and oxidative stress (Gill et al. 2010). Our findings demonstrated an upregulation of TLR-4 expression in the prostates and seminal vesicles of the BPH group, which is supported by previous research (He et al. 2016). TLR4 promotes the EMT process and also activates NADPH oxidase augmenting ROS production (He et al. 2016; Liu et al. 2021; van der Post et al. 2021). Canonical TLR-mediated signaling stimulates NF-κB, a key gene mediator in controlling the inflammatory response, leading to the production of different inflammatory cytokines such as IL-1β (Park et al. 2004; Raafat et al. 2022). The observed elevation of IL-1β in the BPH group aligns with its established role in promoting prostate hyperproliferation, primarily through the stimulation of insulin-like growth factor (IGF) production (Jerde And Bushman 2009). On the other hand, DHM treatment downregulated the expression of TLR4 and IL-1β in testosterone-treated rats, thereby protecting and halting its hyperplastic effects. The anti-inflammatory role of DHM, via the inactivation of TLR4/NFκB/IL-1β signaling, in different organs is well-documented (Chang et al. 2020; Awad et al. 2022; Wei et al. 2022a, b; Matouk et al. 2023, 2024).

Prostate hyperplasia may result from disrupted homeostasis between prostate cell proliferation and apoptosis. As formerly reported in BPH studies, oxidative stress and inflammation activate Bcl-2 which blocks the apoptotic process and consequently induces cellular proliferation. NO enhances the activity of cycloxygenase-2 (COX-2) that generates proinflammatory mediators, induces Bcl-2 expression, and increases prostatic cell proliferation (Wang et al. 2004; Minciullo et al. 2015).

Furthermore, ROS-mediated DNA damage impairs P53-mediated apoptosis, an essential process to remove genetically unstable or abnormally growing cells. Reduced apoptosis may lead to tissue hyperplasia (Sciarra et al. 2008; Wijerathne et al. 2017). Furthermore, growing evidence associated BPH development with upregulated growth factors as TGF-β1 that induce differentiation of epithelial and stromal cells (Jerde And Bushman 2009; He et al. 2016; Abdel-Naim et al. 2018; van der Post et al. 2021; Raafat et al. 2022). Proliferating cell nuclear antigen (PCNA), a cofactor of DNA polymerase, is correlated to DNA replication and repair. It exists in the cytosol binding to procaspases to counteract their conversion into apoptotic caspases. As such, it has been used as the proliferative index in BPH and prostate cancer (Zhang et al. 2021a, b; Raafat et al. 2022; Rasheed et al. 2023). Here, our results demonstrated an increased expression of PCNA in the prostates and seminal vesicles of testosterone-treated BPH rats, suggesting enhanced cellular proliferation. Interestingly, recent studies have displayed a correlation between IL-1β and PCNA in the pathophysiology of prostatic hyperplasia. The increased expression of PCNA in BPH tissue was paralleled with high levels of IL-1β, which matched our results (Raafat et al. 2022).

Noteworthy, the levels of procaspase-3, which represent the inactive form of caspase-3, the executor of apoptotic cell death, were increased in the BPH group. This finding may be puzzling because increased procaspase-3 levels in prostate glands as well as seminal vesicle tissues were parallel with cell hyperplasia and enlargement instead of enhanced apoptosis. However, recent studies have found that procaspase-3 has interesting non-apoptotic activity, and its levels were paradoxically upregulated in some cancers (Brentnall et al. 2014; Boudreau et al. 2019). Moreover, it was reported that PCNA colonizes procaspase-3 as well as procaspase-9 in neutrophils, thus in turn hindering their activation and inhibiting the apoptotic process (Boudreau et al. 2019; Allen 2024). Our data suggested that enhanced PCNA and procaspase-3 correlate with the hyperplastic changes of prostate cells and diminished apoptosis, culminating in the development of BPH. DHM treatment, however, suppressed the expression of PCNA and procaspase-3 accompanied a decline in cell proliferation. The anti-proliferating effects of DHM contribute to its anticancer potential (Zeng et al. 2014), which was recently reviewed in the literature (Wu et al. 2022). It is worth mentioning that DHM-treated group showed higher values of PCNA and procaspase-3 when compared with the normal control. This indicates that while DHM confers substantial protection against testosterone-induced BPH, it may not completely restore their expression levels to baseline, possibly due to the persistent influence of exogenous testosterone on the induced pathology.

DHM could arrest cellular differentiation through different mechanisms, including neutralizing ROS, attenuating inflammatory cytokines including TNF-α and IL-1β, activating apoptotic mediators, arresting cell cycle, preventing angiogenesis, and blocking epithelial-mesenchymal transition (Wu et al. 2022).

**Fig. 9:**
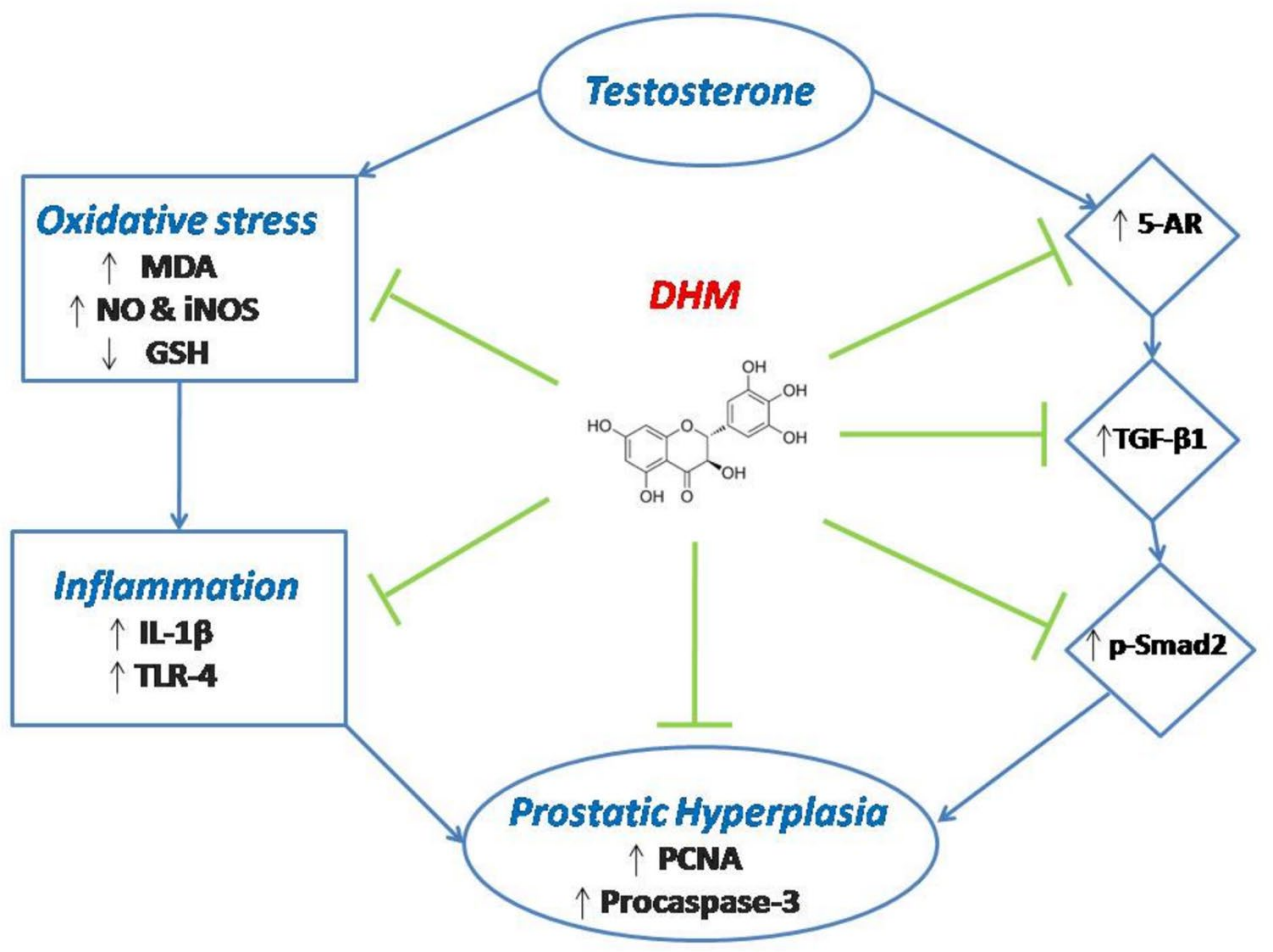
Schematic presentation of the proposed mechanisms of the DHM protective effect against testosterone-induced hyperproliferation in rats

Our suggested mechanisms of the DHM protection against testosterone-induced BPH are summarized in Fig. 9. In brief, DHM exerts its antioxidant activity that leads to a decreased release of several inflammatory mediators. Moreover, DHM mitigates prostatic 5AR-1 expression, which would lead to suppressed expression of TGF-β1 and Smad-2. Both arms end up with decreased prostatic cell hyperproliferation.

A limitation of this study is the lack of assessment of DHM effects on sperm analysis and testicular histology. However, the primary focus of the present study was to elucidate the protective effects of DHM against testosterone-induced proliferation in prostate and seminal vesicle tissues. Future research, including sperm parameters and testicular histology, will be considered to provide a more comprehensive profile of the DHM’s reproductive effect.

## Conclusion

In summary, DHM has shown promising protective effects against testosterone-induced BPH. The anti-BPH effects of DHM were mediated via its antioxidant effects and attenuation of TLR4/IL-1β inflammatory signaling pathway as well as enhancing hormonal balance and reduced serum PSA. DHM ameliorated cellular hyperplasia by suppressing 5α reductase-1 level and inhibiting TGF-β 1/Smad-2 signaling pathway. It also downregulated PCNA and procaspase-3 expression. Additionally, DHM alleviated the testosterone-induced hyperproliferation in seminal vesicle tissues. Therefore, DHM may hold therapeutic potential for androgen-induced hyperplasia via its anti-proliferative, anti-inflammatory, and antioxidant activities.

## Data Availability

The datasets analyzed during this study are available from the corresponding author on request.

## Abbreviations

5-DHT: 5-Dihydrotestosterone
AR: Androgen receptors
BPH: Benign prostatic hyperplasia
DHM: Dihydromyricetin
EMT: Epithelial-mesenchymal transition
GSH: Glutathione
IL-1β: Interleukin-1 beta
iNOS: Inducible nitric oxide synthase
LUTS: Lower urinary tract syndrome
MDA: Malondialdehyde
NO: Nitric oxide
PCNA: Proliferating cell nuclear antigen
PSA: Prostate-specific antigen
ROS: Reactive oxygen species
TGF-β1: Transforming growth factor beta-1
TLR-4: Toll-like receptor 4
